# Effects of Amateur Musical Experience on Categorical Perception of Lexical Tones by Native Chinese Adults: An ERP Study

**DOI:** 10.3389/fpsyg.2021.611189

**Published:** 2021-03-15

**Authors:** Jiaqiang Zhu, Xiaoxiang Chen, Yuxiao Yang

**Affiliations:** ^1^School of Foreign Languages, Hunan University, Changsha, China; ^2^Foreign Studies College, Hunan Normal University, Changsha, China

**Keywords:** amateur musical experience, categorical perception, Mandarin lexical tones, MMN, cortical plasticity

## Abstract

Music impacting on speech processing is vividly evidenced in most reports involving professional musicians, while the question of whether the facilitative effects of music are limited to experts or may extend to amateurs remains to be resolved. Previous research has suggested that analogous to language experience, musicianship also modulates lexical tone perception but the influence of amateur musical experience in adulthood is poorly understood. Furthermore, little is known about how acoustic information and phonological information of lexical tones are processed by amateur musicians. This study aimed to provide neural evidence of cortical plasticity by examining categorical perception of lexical tones in Chinese adults with amateur musical experience relative to the non-musician counterparts. Fifteen adult Chinese amateur musicians and an equal number of non-musicians participated in an event-related potential (ERP) experiment. Their mismatch negativities (MMNs) to lexical tones from Mandarin Tone 2–Tone 4 continuum and non-speech tone analogs were measured. It was hypothesized that amateur musicians would exhibit different MMNs to their non-musician counterparts in processing two aspects of information in lexical tones. Results showed that the MMN mean amplitude evoked by within-category deviants was significantly larger for amateur musicians than non-musicians regardless of speech or non-speech condition. This implies the strengthened processing of acoustic information by adult amateur musicians without the need of focused attention, as the detection of subtle acoustic nuances of pitch was measurably improved. In addition, the MMN peak latency elicited by across-category deviants was significantly shorter than that by within-category deviants for both groups, indicative of the earlier processing of phonological information than acoustic information of lexical tones at the pre-attentive stage. The results mentioned above suggest that cortical plasticity can still be induced in adulthood, hence non-musicians should be defined more strictly than before. Besides, the current study enlarges the population demonstrating the beneficial effects of musical experience on perceptual and cognitive functions, namely, the effects of enhanced speech processing from music are not confined to a small group of experts but extend to a large population of amateurs.

## Introduction

Pertaining to the old relationship between music and language, it is believed that the spoken language evolves from music (Darwin, 1871), or music evolves from the spoken language (Spencer, 1857), or both of them descend from a common origin (Rousseau, 1781/1993). These viewpoints bolster the notion that music and language, both of which involve complex and meaningful sound sequences (Patel, 2008), are reciprocally connected. It has been considerably verified that musical experience impacts on language or speech processing (Besson et al., 2011a,b, Patel, 2014). One of the most common approaches to the assessment of speech processing is adopting categorical perception in experiments, in which individuals are required to perceptually categorize continuous auditory signals into discrete linguistic representations along a physical continuum (Fujisaki and Kawashima, 1971). The phenomenon of categorical perception has been investigated with preliminary foci on segments of consonants and vowels (e.g., Liberman et al., 1957; Fry et al., 1962; Miller and Eimas, 1996).

In recent years, a surge of interest has been observed concerning the suprasegmental aspect of speech processing (e.g., Yip, 2002; Hallé et al., 2004; Xu et al., 2006; Peng et al., 2010; Liu et al., 2018). One example is the research into lexical tones, which are phonemically contrastive and alter the semantic meanings of words in usage (Gandour and Harshman, 1978). Through shifts in pitch height and pitch contour, lexical tones can be distinguished and recognized in disparate categories (Francis et al., 2003). For instance, monosyllables “ma 55,” “ma 35,” “ma 214,” and “ma 51” in Mandarin mean “mother,” “hemp,” “horse,” and “to scold,” when presented individually (Chao, 1968). The numerals following the syllables stand for the transcribed tones, depicting the relative pitch value within a five-point scale of the talker’s normal frequency range (Chao, 1947). These four tones can also be annotated with respective pitch patterns as Tone 1 (T1), level; Tone 2 (T2), rising; Tone 3 (T3), falling-rising; and Tone 4 (T4), falling (Wang et al., 2017).

### Categorical Perception of Lexical Tones

Lexical tones are privileged in Mandarin both phonetically and phonologically, and categorical perception of lexical tones has been broadly researched in present decades (Xu et al., 2006; Peng et al., 2010; Chen F. et al., 2017). In a classic experiment of categorical perception, participants complete an identification task to label a flow of tonal stimuli and a discrimination task to estimate some tonal contrasts as either “same” or “different” (Xu et al., 2006). The auditory stimuli are physically interpolated with variable pitch values along a continuum (Francis et al., 2003). Separated by categorical boundary between two tones as defined by identification curves (Peng et al., 2010), within-category stimuli stemmed from one category can be perceived analogously, but across-category stimuli extracted from two categories tend to be perceived differentially. Stimulus tokens from discrepant categories are more discriminable than those from the same category (Joanisse et al., 2006; Jiang et al., 2012; Chen and Peng, 2018).

Most of prior studies probed into lexical tones as a whole (e.g., Wang et al., 2001; Francis et al., 2003; Hallé et al., 2004), while more recent studies have switched to the dynamic interaction between acoustic and phonological information of lexical tones (e.g., Xi et al., 2010; Zhang et al., 2011; Yu et al., 2014). In general, the acoustic information consists of the physical features of lexical tones as estimated by F0 (e.g., pitch height and pitch contour), while the phonological information refers to the linguistic properties with tonal categories to distinguish lexical semantics (Yu et al., 2019). Although some secondary cues might influence the judgment of lexical tone contrasts, F0 remains most critical as amply confirmed by the seminal (Wang, 1976) and subsequent studies of categorical perception of lexical tones (Xu et al., 2006; Peng et al., 2010; Shen and Froud, 2016). It is reported that for Mandarin lexical tones, the perception of within-category pairs mainly depends on lower-level acoustic information of pitch, yet the perception of across-category comparisons is principally reliant on higher-level phonological information of lexical categories (Fujisaki and Kawashima, 1971; Xi et al., 2010; Yu et al., 2014). Importantly, distinguishing acoustic and phonological information of lexical tones re-paints a clear picture to interpret the mechanisms underlying lexical tone perception. For example, the study by Xi et al. (2010) revealed that when listening to Mandarin lexical tones, native speakers need to process the acoustic information and the phonological information simultaneously. A following study by Yu et al. (2014) manipulated phonological categories and acoustic intervals of lexical tones in experiments and replicated the results of Xi et al. (2010). Their additional findings uncovered the temporal pattern of lexical tone processing showing that phonological processing precedes acoustic processing at the pre-attentive cortical stage, which was then re-confirmed in a subsequent study by Yu et al. (2017). The pre-attentive stage refers to an earlier stage at which individuals involuntarily process the stimuli, in contrast to the later attentive stage at which individuals consciously process the stimuli (Neisser, 1967; Kubovy et al., 1999; Yu et al., 2019). According to Zhao and Kuhl (2015a, b), this pattern of temporal processing involves the dominant influences of the higher-level linguistic categories as compared to the lower-level acoustics relating to lexical tones.

Given that native speakers of tone languages outperform those of non-tone languages, a plethora of studies support the notion that lexical tone perception is plastic and experience-dependent (Hallé et al., 2004; Peng et al., 2010; Shen and Froud, 2016; Chen S. et al., 2017). To be exemplified, compared to Mandarin speakers who perceive native lexical tones categorically, participants from non-tone languages show impoverished performance in tasks requiring identification and discrimination of lexical tones. In the study by Hallé et al. (2004), although French listeners were observed to have substantial sensitivity to pitch contour differences, they failed to perceive lexical tones along the lines of a well-defined and finite set of linguistic representations as exhibited by across-category tonal contrasts. However, those speaking the tone language Taiwanese could perceive lexical tones in a quasi-categorical manner. The authors proposed that this disparity was attributable to the existence of phonological information relating to lexical tones in Taiwanese, which was, however, absent in French. Considering the gradation of identification and discrimination tasks, Peng et al. (2010) demonstrated that German listeners exhibited larger boundary widths and psychophysical boundaries rather than linguistic boundaries compared to their Mandarin and Cantonese counterparts. The results of Xu et al. (2006) also exhibited strong categorical perception for Chinese-speaking but not for English-speaking participants in their experiments. According to Chen et al. (2020), listeners from non-tone languages exhibited psychoacoustically based performance because of the lack of experience with lexical tones; however, Shen and Froud (2016) found that with increased exposure to lexical tones, English learners of Mandarin would show similar performance to native Mandarin speakers. Since categorical perception provides an ideal window to disentangle acoustic information from phonological information through pairing respective within- and across-category auditory stimuli, the current study would make use of this paradigm to research the processing of these two types of information, which is less studied among listeners with different levels of musical pitch expertise.

### The Influence of Musicianship on Lexical Tone Processing

The brain perceptual plasticity of lexical tones induced by language experience has been further demonstrated via cross-domain research, suggesting that musical experience also impacts on the perception of lexical tones. Besson et al. (2011a) proposed that the musician’s brain is a good model of brain plasticity. The links between music and language are grounded on findings from numerous empirical studies (e.g., Schön et al., 2004; Marques et al., 2007; Moreno, 2009; Posedel et al., 2012; Ong et al., 2020). It is believed that musicians show advantages in processing and encoding speech sounds due to increased plasticity and perceptual enhancements (Magne et al., 2006; Kraus and Chandrasekaran, 2010; Gordon et al., 2015). According to the expanded hypothesis of Overlap, Precision, Emotion, Repetition and Attention (OPERA-e), musical experience enhances speech processing because common sensory and cognitive processing mechanisms are shared by music and language (Patel, 2014). Based on the conceptual framework of OPERA-e, the perception of fine-grained musical pitches remains transferrable to that of coarse-grained lexical tones, as supported by the studies on music and lexical tone processing. For example, Alexander et al. (2005) found that a group of American musicians obtained higher scores than non-musicians in identifying and discriminating four lexical tones in Mandarin. Zhao and Kuhl (2015a) demonstrated that, given no prior experience of Mandarin or any other tone languages, English-speaking musicians were more sensitive to pitch variations of tonal stimuli from Mandarin T2–T3 continuum than non-musicians. A follow-up experiment of perceptual training of lexical tones was held, results demonstrating that compared to the non-musician counterparts, musician trainees showed improvement in identification in post-training test (Zhao and Kuhl, 2015a). It certified that short-term perceptual training altered perception, which spanned only about 2 weeks. The study by Moreno et al. (2008) investigated individuals trained via respective music and painting lessons. The results uncovered that participants with musical training were strengthened in both pitch discrimination and reading abilities, in contrast to those with painting training. It highlighted the influence of musical experience on speech processing. Zheng and Samuel (2018) also found that English-speaking musicians outperformed non-musicians in the processing of both speech (Mandarin short phrases) and non-speech (F0) sounds.

The research into music and lexical tone processing has benefitted from neurophysiological assessments applied in an array of studies, which allow monitoring the perception of auditory signals within the brain. One of the most prevalent approaches is the measurement of event-related potentials (ERPs) to quantify brain activities in response to specific events with a high-temporal resolution in millisecond. Because the auditory ERP component, known as the mismatch negativity (MMN), can evaluate automatic discrimination at the pre-attentive cortical stage, it has been pervasively employed in neural studies of lexical tone perception (e.g., Chandrasekaran et al., 2007a,b; Li and Chen, 2015; Nan et al., 2018). Specifically, MMN peak latency reflects the time course of cognitive processing, while MMN mean amplitude indexes the extent to which neural resources relate to our brain activities (Duncan et al., 2009). Through ERP measurements, Besson et al. (2011a) tested the perception of Mandarin tonal and segmental variations among French-speaking musicians and non-musicians. The results exhibited increased amplitude and shorter latency of ERP components of N2, N3, and P3 for musicians than non-musicians, suggesting that musical expertise impacts on the categorization of foreign linguistic contrasts. Chandrasekaran et al. (2009) examined the perception of non-speech tone homologs to Mandarin T1, T2, and a linear rising ramp (T2L). The results revealed that English-speaking musicians provoked larger MMN responses than their non-musician counterparts, regardless of within-category (T2/T2L) or across-category (T1/T2) tonal contrast. This finding indicates that experience-dependent effects of pitch processing are domain-general. In spite of the preexisting long-term experience of lexical tones, Tang et al. (2016) found that Mandarin-speaking musicians showed increased MMN amplitude to changes of lexical tones compared to non-musicians, which implicates that musical experience facilitates cortical plasticity of linguistic pitch processing.

Although the studies regarding the influence of musical experience on lexical tone processing are not rare, most concentrate on the performance of professional musicians, who usually complete long-term musical training for tens of years (e.g., Pantev et al., 2001; Marie et al., 2012; Dittinger et al., 2016), receive formal and theoretical musical education in music conservatories (e.g., Vuust et al., 2012; Lee et al., 2014; Tang et al., 2016), and start musical practice very early (before puberty) in life (e.g., Cooper and Wang, 2012; Zhao and Kuhl, 2015a,b). In stark contrast to expert musicians in a preponderance of studies mentioned above, amateur musicians involve those who are non-music majors with later onset age and shorter musical length for their limited musical experience. Since it has been widely accepted that music plays a strong modulatory role in boosting language or speech processing (Patel, 2008, 2014; Besson et al., 2011a,b; Strait and Kraus, 2014), a question then arises as to whether individuals with amateur musical experience can obtain similar advantageous effects to experts such as in categorical perception of lexical tones.

A very recent study identified that children who attended informal musical group activities demonstrated better neural sound discrimination than controls (Putkinen et al., 2019). However, it remains less known whether the facilitative effects of amateur musical experience can also be found in adults, who diverge from children in light of physiological maturation and language exposure. Gfeller (2016) argued that both music and language as communicative forms encompass many subskills, and these are impacted by maturation as well as auditory input and experience (Yang et al., 2020). In the study by Chen et al. (2010), prelingually deafened participants with cochlear implants completed the pitch ranking of tonal pairs. The results showed that the length of musical experience was beneficial only for young participants. Best (1994, 1995) and Best and Tyler (2007) concluded that children and adults performed differently in speech perception because their perceptual systems had been tuned to variable degrees as a function of native language exposure. For example, according to Best et al. (2016), the discriminability of two phones produced with the same articulatory organ improves with increased native language exposure, yet the same improvement was not observed among adults in Yang and Chen (2019). In addition, for Mandarin-speaking participants, the significant differences in voice onset time, defined as the time interval of the burst and the beginning of glottal pulse in stop consonants (Cho and Ladefoged, 1999), were observed in Ma et al. (2017), which were ascribed to the physiological differences between children and adults. Given the vast disparities in neuroplasticity and hearing history, adults and children might have different bioelectrical responses to auditory stimuli at the pre-attentive stage originating from the impact of amateur musical experience.

### The Present Study

The study by Putkinen et al. (2019) serves as an encouraging indication of the benefits of musical exposure while highlighting the need to continue researching the effects of amateur musical experience throughout adulthood. We recruited adult participants with limited musical experience (mean ± standard deviation: 4.5 ± 0.3, range: 4–5 years) of playing orchestral instruments requiring intensive usage of musical pitch (Sluming et al., 2002; Vuust et al., 2012; Patel, 2014). Although these participants reported that they always enjoyed their musical practice, none of them had received an early musical education, taken private lessons, or obtained any professional certificates in musical practice; crucially, their involvement in music (all after 16 years old) was motivated by self-willingness rather than commercial performance (Marie et al., 2012). The musical expertise of these amateur musicians in this study was prominently lower than that of expert musicians investigated in previous studies (e.g., Pantev et al., 2001; Alexander et al., 2005; Vuust et al., 2012; Wu et al., 2015; Zhao and Kuhl, 2015a,b).

What has been unveiled thus far about adult amateur musicians is sparse; however, the population of adult amateur musicians worldwide is enormous, in contrast to the limited number of professional musicians. The investigation of the effects of amateur musical experience on adults’ perception of lexical tones is of great importance, because it helps resolve the research issues mentioned above and address whether participants with amateur musical experience should be differentially grouped from non-musicians, when conducting experiments in relation to the processing of word-level lexical tones or sentence-level intonations (Qin et al., 2021). That is to say, among these tests, it might be inappropriate to ignore participants’ musical expertise or simply regard those with informal musical training as non-musicians. In addition, both behavioral and neural studies have elucidated that musicianship brings positive impacts on language and cognition across the life span for children (Schellenberg, 2004), adults (Wang et al., 2015), and aging citizens (Román-Caballero et al., 2018). Therefore, findings from the current study might encourage more individuals to participate in musical activities no matter what levels of performance they maintain and what backgrounds they are from, in the hope of increasing their aesthetic appreciation as well as helping them balance their physical and mental health.

In the current study, categorical perception of lexical tones was adopted so as to tease apart acoustic and phonological information by pairing respective within- and across-category stimuli (Yu et al., 2014). Except for the investigation of lexical tones in the speech condition, pure tones as the non-speech stimuli with congruous F0 features were meanwhile exploited to examine whether music-driven and experience-dependent effects of pitch processing were domain-general (Chandrasekaran et al., 2009). Grounded on the framework of OPERA-e (Patel, 2014) that common sensory and cognitive processes relating to pitch permit the facilitative effects from music to lexical tone perception, discrepancies were anticipated between amateur musicians and non-musicians with respect to the coverage of MMN mean amplitudes and MMN peak latencies. Concretely, according to preceding studies (Chandrasekaran et al., 2009; Besson et al., 2011a; Wu et al., 2015; Tang et al., 2016), MMN mean amplitudes were expected to be larger in the perception of within-category stimuli by amateur musicians than non-musicians, yet both of them would be comparable when processing across-category stimuli; in other words, amateur musicians might be only enhanced in acoustic processing for native lexical tones. As to MMN peak latencies, there exist two competing views about the time course of acoustic and phonological processing of lexical tones. Luo et al. (2006) proposed a serial model, the two-stage model, arguing that only acoustic information of lexical tones is processed at earlier pre-attentive stage and phonological information is processed at later attentive stage. However, many recent ERP studies showed that phonological information of lexical tones is processed in parallel with acoustic information at the pre-attentive stage (Xi et al., 2010; Yu et al., 2014, 2017). In this regard, we hypothesized that acoustic and phonological information would be processed concurrently, which contradicted the two-stage model of lexical tone processing (Luo et al., 2006).

To the best of our knowledge, the present study is the first attempt to clarify the aforementioned issues in Chinese adult population from a neural perspective. By exploring whether the facilitative effects from music to speech processing could be grasped by a large group of amateur musicians similar to experts in previous studies, this study aimed to provide an in-depth understanding of neuroplasticity in addition to the processing of lexical tones after re-visiting the relationship between music and language.

## Materials and Methods

### Participants

Thirty adult Mandarin-speaking college students (18 males and 12 females, aged 21–30 years, mean age 24) were recruited from universities in Shenzhen, China through online advertising. All participants were confirmed as having no history of speech or hearing disorders, learning disabilities, brain injuries, or neurological problems (experienced themselves or by relatives). Based on the well-documented criteria for classifying musical expertise (e.g., Pantev et al., 2001; Alexander et al., 2005; Marie et al., 2012; Hutka et al., 2015), the participants were divided into two groups: non-musicians (NM) and amateur musicians (AM). The AM group consisted of 15 amateur musicians (6 males and 9 females, aged 21–25 years, mean age 23), none of whom majored in music. Their limited musical experience ranged from 4 to 5 years with the mean and standard deviation at 4.5 and 0.3, individually. The NM group served as the control group, which consisted of 15 participants (12 males and 3 females, aged 22–30 years, mean age 24) with no musical experience (e.g., playing an instrument or vocal training). Although no power analysis was performed for the calculation of sample size, the sample size of the current study was comparable with one seminal ERP study by Xi et al. (2010) that also focused on the processing of acoustic versus phonological information via categorical perception of Mandarin lexical tones. All participants were paid monetarily for their participation.

Both AM and NM members were right-handed according to a handedness questionnaire adapted from a modified Chinese version of the Edinburgh Handedness Inventory (Oldfield, 1971). Consent forms were signed by participants prior to the experiment, which was approved by the Ethics Review Board at the School of Foreign Languages of Hunan University.

### Stimuli

Sampled at 44.1 kHz and digitized at 16 bits, the Chinese monosyllable /pa/ was recorded with respective T2 and T4 in a sound-attenuated room by a native female speaker from northern Mainland China. The primary cue to distinguish tonal contrasts in Mandarin refers to pitch, known as the psychological percept of F0 (Abramson, 1978; Gandour, 1983). Nevertheless, the comparison of some pairs of Mandarin lexical tones may be affected by cues in addition to pitch. For example, distinguishing T2 and T3 always confuses both native and non-native Mandarin speakers (Li and Chen, 2015), and the timing of the turning point in pitch contour is also critical for the discrimination (Shen and Lin, 1991). The signal properties of T2 and T3 are not very distinctive and their acoustic similarities are further compounded by the Mandarin tone sandhi (Hao, 2012). Unlike similar tones of T2 and T3, T2 and T4 have disparate pitch contours and remain phonologically distinctive (Chao, 1948). Hence, Mandarin T2 and T4 were purposefully selected, not only because they had been appreciably employed in categorical perception of lexical tones in previous studies, but also because the discrimination of T2 and T4 was reliant on the detection of pitch variations rather than other confounding features (e.g., phonation) in the acoustic signals (Xi et al., 2010; Zhang et al., 2011; Li and Chen, 2015; Zhao and Kuhl, 2015a). The examination of T2 and T4 was likely to maximize the potential differences between amateur musicians and non-musicians in the processing of across-category and within-category stimuli along the tonal continuum.

**FIGURE 1:**
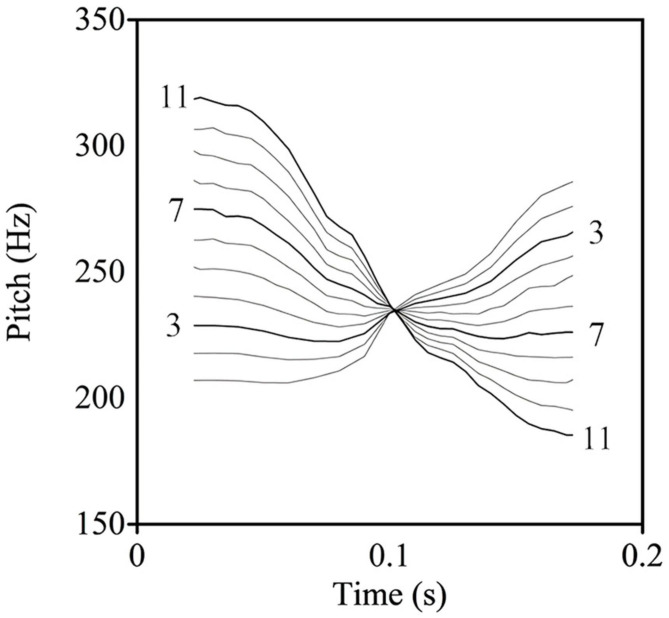
The schematic illustration of the tonal continuum (the thick lines with numerals represent the stimuli used in the neural tests).

The two lexical tones were normalized to a sound pressure level of 70 dB and a duration of 200 ms using the Praat software (Boersma and Weenink, 2019). In addition, the Mandarin T2–T4 continuum was manipulated by applying the pitch-synchronous overlap and added function (Moulines and Laroche, 1995) via Praat. As shown in Figure 1, 11 stimulus sets were created spanning the continuum with an equalized acoustic interval between each step. Prototypically, the first stimulus (S1) referred to T2 and the last stimulus (S11) signaled T4. The non-speech stimuli were pure tones, with exactly the same pitch, intensity, and duration as the speech stimuli, which were resynthesized following the procedures of Peng et al. (2010).

According to prior studies on categorical perception, the third (S3) and the last (S11) stimuli were chosen as deviants, with the seventh (S7) being the standard from the current continuum, which would be played in the neural tests (Xi et al., 2010; Zhang et al., 2011; Yu et al., 2014). Although both deviants were equidistant in frequency size to the standard, the stimuli of S3 and S11 were defined as across-category and within-category deviants, respectively. Crucially, to further assure the feasibility of stimulus deployment, an identification task was conducted in order to locate the categorical boundary (Peng et al., 2010). The categorical boundary was computed using Probit analysis, which involved the commensurate 50% crossover point in the continuum (Finney, 1971). Based on the boundary position, across-category and within-category stimuli for each participant could be paired in agreement with Jiang et al. (2012). For instance, if one participant retained the boundary position as 4.9 in the identification task, pairs S3–S5 and S4–S6 that straddled the position would be coded as across-category comparisons, whereas the remaining pairs that did not cross the boundary would be taken as within-category comparisons (Chen F. et al., 2017).

**FIGURE 2:**
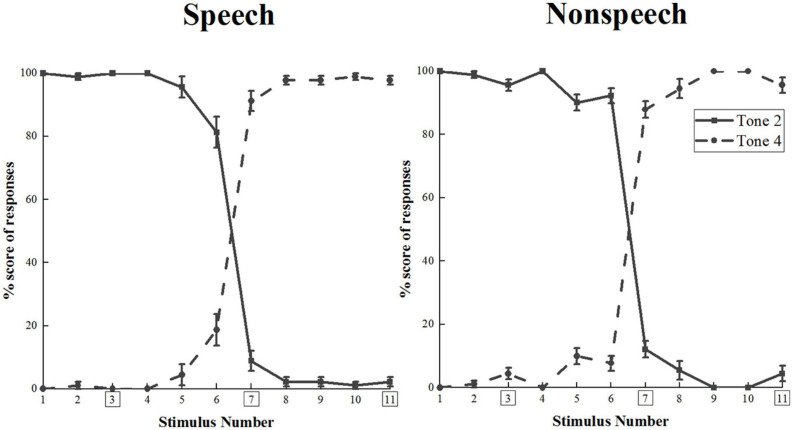
The identification curves for the speech and non-speech stimuli among native Chinese adults (vertical bars represent one stand error). T2 and T4 were coded as S1 and S11; besides, S3, S7 and S11 with rectangles represented across-category deviant, standard and within-category deviant stimuli, respectively.

The identification task was completed by 10 participants who did not attend the following electroencephalogram (EEG) recording phase. All 11 stimuli were presented randomly through a laptop using the E-Prime 2.0 program (Psychology Software Tools Inc., United States). Each stimulus was played nine times. The design of two-alternative forced choices was applied, thereby participants had to make a choice when they heard the sounds. Both T2 and T4 were labeled on the keyboard and participants pressed the target buttons to respond. Figure 2 demonstrates the identification curves.

In the current study, categorical boundary positions in speech and non-speech conditions were 6.47 and 6.46, respectively, which indicated that the pairing of S3–S7 was across-categorical, while S7–S11 remained within-categorical. Therefore, as mentioned above, the present stimulus deployment was operationalized and could be applied to the next EEG data collection. In addition, for both AM and NM participants, one more active behavioral identification task was carried out after their recordings of ERPs. All participants correctly identified the three lexical tones as either T2 or T4 with 0% error rate out of the four choices from T1, T2, T3, and T4 in Mandarin. The results revealed that amateur musicians and non-musicians perceived S3–S7 as an across-category comparison and S7–S11 as a within-category comparison.

### ERP Procedure

**FIGURE 3:**
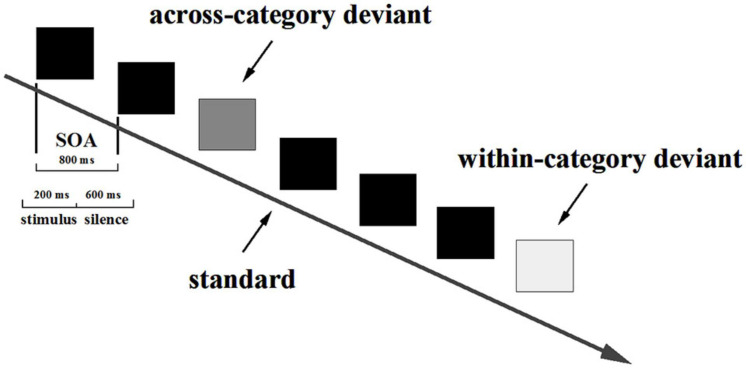
The schematic diagram for typical trials in the improved passive oddball paradigm.

In line with Näätänen et al. (2004) and Pakarinen et al. (2007), the current study adopted a multifeature passive oddball paradigm, which consists of more than one type of deviant in one block (Partanen et al., 2013; Yu et al., 2019). The 15 standard stimuli were played first to prompt participants to establish a standard perceptual template. Then, 1,000 stimuli (800 standards and 200 deviants) were played binaurally. The number of each type of deviant was 100. The stimulus-onset asynchrony (SOA) was 800 ms, and each sound was presented for 200 ms. The deviants were repeated pseudo-randomly with any two adjacent deviants separated by at least three standards, as displayed in Figure 3. The speech stimuli were set into one block and the non-speech stimuli were contained in another block. Two blocks were presented for all participants in a counterbalanced sequence. The whole experiment lasted around 1 h, including a 5-min break between blocks and a 10-min show of a movie before the tests.

The experiment was conducted in an acoustically and electrically shielded chamber. The participants were seated in front of two active loudspeakers placed to their right and left side with a 45° angle, both of which were kept 0.5 m distance from their ears. An electronic tablet was provided for the participants to play a movie that none had already watched to distract their attention from the sounds. Although the movie was kept silent, the subtitles appeared as normal. The participants were told that they should watch the movie carefully in the whole process because questions would be asked about the movie before and after the EEG recording. For example, prior to the EEG recording, the participants needed to answer one question after viewing the movie. When the right answer was provided, the formal EEG recording would proceed during which participants were instructed to minimize head motion and eye blinking while sitting quietly in the reclined chair.

### EEG Recording

An EGI GES 410 system with 64 channel HydroCel GSN electrode nets was employed for the EEG data collection. The vertex (Cz) was settled as the reference electrode when the continuous EEG data were recorded. The vertical and horizontal electrooculograms were monitored by the electrodes placed on the supra- and infra-orbital ridges of each eye and the electrodes near the outer canthi of each eye, respectively. The data were digitized at 1 kHz and amplified with a band-pass filter of 0.5–30 Hz. The impedance of each contact channel was maintained below 50 kΩ (Electrical Geodesics, 2006).

### Data Analysis

The EEG data were analyzed off-line with custom scripts and EEGLAB running in the MATLAB environment (Mathworks Inc., United States). With re-reference to the average of all electrodes, the data were adjusted by eliminating the interference of horizontal and vertical eye-movements. The recordings were off-line band-pass filtered with 1–30 Hz and segmented into a 700-ms time window with a 100-ms pre-stimulus baseline. The baseline was then corrected and the recorded trials with ocular or movement artifacts were rejected if they exceeded the range of −50 to 50 μV. Only those data with at least 80 accepted deviant trials for each deviant type were used. The ERPs elicited by standard and deviant stimuli were computed on average of trails of each participant, whereby the MMNs were obtained through the deviant-minus-standard formula.

Consistent with the extant literature, three recording sites of F3, F4, and Fz were selected for statistical analysis (Xi et al., 2010). The time window for MMN typically peaks around 200–350 ms based on the studies by Näätänen et al. (1978, 2007). As shown by Yu et al. (2014), there exist multiple time windows for MMN in different experiments, such as 100–350 ms, 150–300 ms, and 230–360 ms. In line with a recent study by Luck and Gaspelin (2017), an approach termed “Collapsed Localizers” (which is becoming increasingly common) was applied to identify the current time window for MMN^1^. The MMNs were firstly obtained by subtraction of ERP waveforms of the standard from those of the deviants for both conditions. After obtaining MMNs, these difference waveforms were averaged across all participants (AM and NM) and conditions (speech and non-speech), whereby the collapsed waveform was unbiasedly inspected (without showing group and condition differences). In the present study, the time window for MMN was fixed at 100–300 ms based on this collapsed waveform. The MMN mean amplitude was computed as the mean voltage from the range of 20 ms before and after the MMN peak at Fz. The statistical analyses of MMN mean amplitude and MMN peak latency were implemented on the three chosen recording electrodes (F3, F4, and Fz).

## Results

**FIGURE 4:**
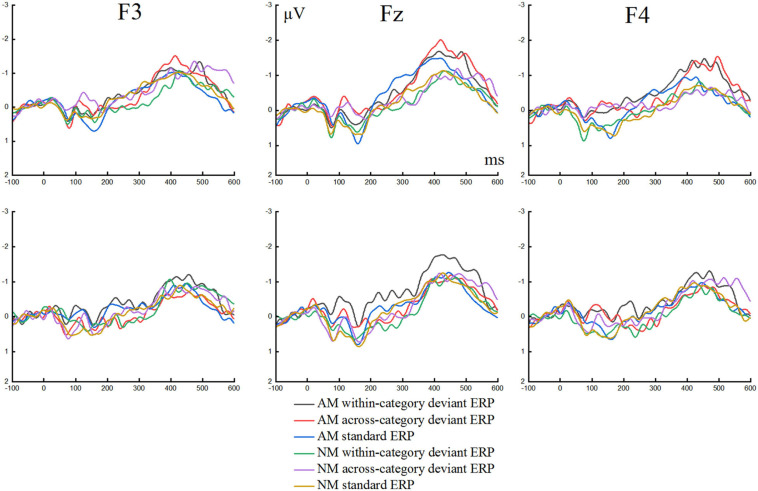
Grand average waveforms elicited by standard and deviant stimuli in speech condition **(the upper row)** and non-speech condition **(the lower row)** at three electrodes for amateur musicians (AM) and non-musicians (NM).

**FIGURE 5:**
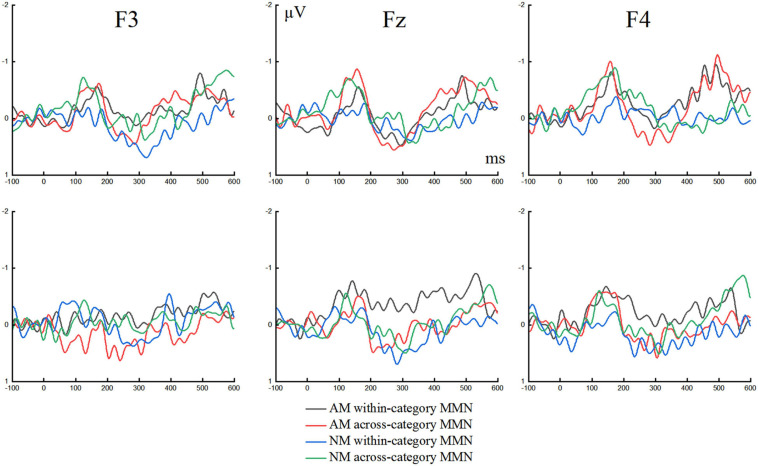
The difference waveforms evoked by across-category and within-category changes in speech condition **(the upper row)** and non-speech condition **(the lower row)** at three electrodes for amateur musicians (AM) and non-musicians (NM).

The grand average waveforms of the ERPs elicited by the standard and deviant stimuli in speech and non-speech conditions at three locations of F3, F4, and Fz are presented in Figure 4. The MMNs obtained via deviant-minus-standard formula of the ERPs for both conditions at F3, F4, and Fz are portrayed in Figure 5. Two three-way repeated measures analyses of variance (ANOVAs) were conducted for MMN peak latency and MMN mean amplitude, respectively, with Condition (speech and non-speech) and Deviant type (within-category and across-category stimuli) as two within-subject factors, and Group (AM and NM) as the between-subject factor. For all analyses, the degrees of freedom were adjusted according to the Greenhouse–Geisser method.

### MMN Mean Amplitude

**FIGURE 6:**
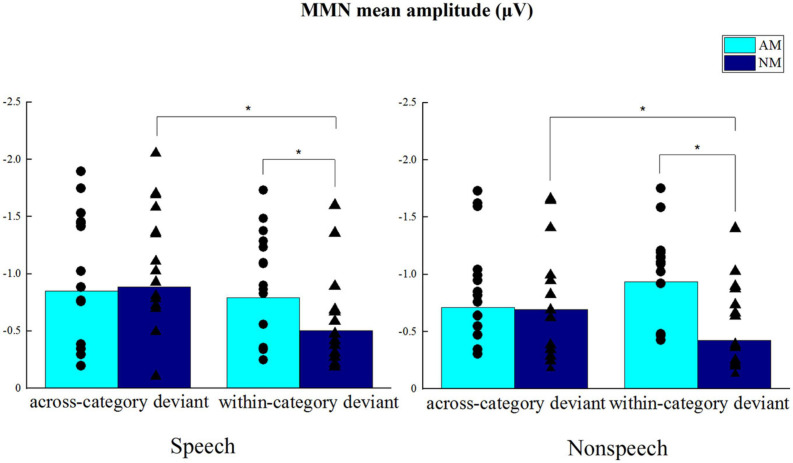
MMN mean amplitudes from electrodes F3, F4, and Fz in respective speech and non-speech conditions. *p < 0.05.

The MMN mean amplitudes are shown in Figure 6, which presents the clear differences between AM and NM groups in the processing of within-category deviants. It can be seen that the differences become less pronounced when both groups perceived across-category deviants. ANOVA indicated that the main effect of Group was not significant, *F*(1,28) = 1.875, *p* = 0.182, the main effect of Condition was not significant, *F*(1,28) = 0.243, *p* = 0.626, and the main effect of Deviant type was not significant, *F*(1,28) = 1.425, *p* = 0.243. However, a marginally significant interaction between Deviant type and Group was yielded, *F*(1,28) = 3.962, *p* = 0.056. Further simple effects analysis for this interaction revealed that, regardless of speech or non-speech condition, the AM group showed significantly larger MMN mean amplitude than the NM group in the processing of within-category deviants, *F*(1,28) = 5.211, *p* < 0.05. For across-category deviants, there was no significant difference between the two groups in terms of MMN mean amplitude, *F*(1,28) = 0.004, *p* = 0.951. Moreover, for the NM group, MMN mean amplitude evoked by across-category stimuli was significantly larger than that by within-category stimuli, *F*(1,28) = 5.07, *p* < 0.05, but there was no significant difference between deviant types for the AM group, *F*(1,28) = 0.317, *p* = 0.578. The interaction between Deviant type, Condition and Group was not significant, *F*(1,28) = 0.176, *p* = 0.678. Taken together, the results of MMN mean amplitude confirmed that amateur musicians were enhanced in processing within-category deviants and more sensitive in detecting pitch shifts, evidenced through their larger MMN mean amplitude, as compared to non-musicians.

### MMN Peak Latency

**FIGURE 7:**
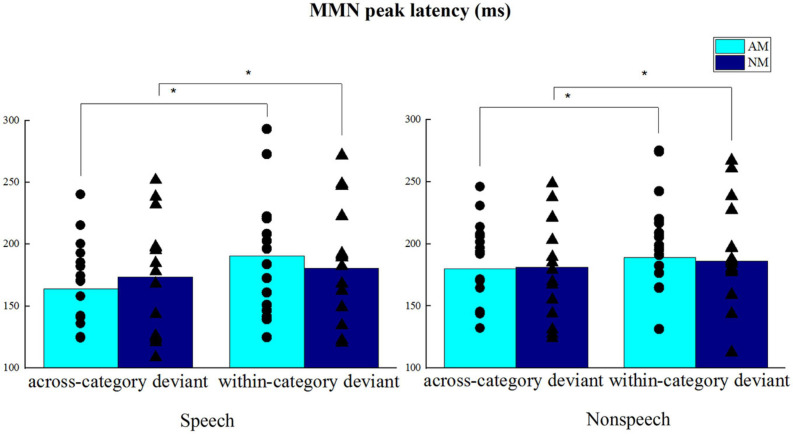
MMN peak latencies from electrodes F3, F4, and Fz in respective speech and non-speech conditions. *p < 0.05.

The MMN peak latencies are displayed in Figure 7, which shows the clear differences between across-category and within-category deviants in the speech condition, whereas the distinctions are less prominent in the non-speech condition. ANOVA revealed that the main effect of Group was not significant, *F*(1,28) = 0.002, *p* = 0.961, the main effect of Condition was not significant, *F*(1,28) = 0.551, *p* = 0.464, but there was a significant main effect of Deviant type, *F*(1,28) = 6.428, *p* < 0.05, across-category deviant < within-category deviant. No significant interaction was found between Deviant type and Group, *F*(1,28) = 1.618, *p* = 0.214, and no significant three-way interaction was found between Deviant type, Condition and Group, *F*(1,28) = 0.381, *p* = 0.542. Meanwhile, the other effects did not reach statistical significance (*p*s > 0.1). The results of MMN peak latency indicated that both amateur musicians and non-musicians perceived across-category deviants earlier than within-category deviants at the pre-attentive cortical stage.

## Discussion

Results of the ERP measurements indicated that both AM and NM groups provoked the significantly shorter MMN peak latency for across-category stimuli than within-category stimuli, which partially certifies our hypotheses of latency showing that not only these two types of information were processed concurrently but phonological information was processed prior to acoustic information of lexical tones at early pre-attentive stage, irrespective of speech or non-speech condition. Meanwhile, the AM group exhibited the significantly larger MMN mean amplitude than the NM group in the processing of within-category deviants in both speech and non-speech conditions, which certifies our hypotheses of amplitude indicative of the AM group’s better automatic discrimination of pitch at the pre-attentive cortical stage. These findings manifest that amateur musicians and non-musicians differ in their MMN profiles, suggesting that perceptual processing of lexical tones by amateur musicians is divergent from that by their non-musician counterparts via distinctive neurocognitive mechanisms. This lends support to the notion that musicianship modulates categorical perception of lexical tones. Besides, an association between language and music, even at an amateur level of musical expertise, is evidenced by the empirical data. Furthermore, alterations in plasticity can also be induced in adulthood, since the facilitative effects from music to linguistic pitch processing appeared for adult native speakers who had preexisting long-term tone language experience. Coming out of the traditional conception, this proves a novel point that the advantageous effects from music to speech processing can be obtained by a large population of amateurs rather than only by a small group of experts.

### Enhanced Acoustic Processing but Comparable Phonological Processing of Lexical Tones Between AM and NM

The results exhibited that amateur musicians provoked the significantly larger MMN mean amplitude when processing within-category deviants than non-musicians independent of speech or non-speech condition, suggesting that amateur musicians were more sensitive to acoustic information of lexical tones. According to the majority of previous studies (Xu et al., 2006; Peng et al., 2010; Yu et al., 2014, 2017; Shen and Froud, 2016), native tone language users mainly decode phonological information when across-category comparisons are heard; nonetheless, the discrimination of within-category comparisons demands fine-grained pitch resolution. The experimental stimuli (S3, S7, and S11) were adopted from the Mandarin T2–T4 continuum. Viewed from the perspective of signal properties, although S3 and S11 both maintained four steps apart from S7, the combinations of S3–S7 and S7–S11 were essentially different in perception for native speakers. For the pairs of S3–S7 and S7–S11, the former tones had straddled the categorical boundary between T2 and T4, yet the latter only belonged to the same category of T4. Therefore, the discrepancies between across-category (S3–S7) and within-category (S7–S11) comparisons influenced perceptual processing differentially. Moreover, considering that other cues, such as duration and intensity, had already been normalized, and the perception of Mandarin T2 and T4 is determined by pitch variations instead of some confounding features (Li and Chen, 2015; Zhao and Kuhl, 2015a), the results that the AM group showed larger MMN mean amplitude in perceiving within-category deviants implied that the AM participants had stronger pitch-processing abilities as compared to their non-musician counterparts.

Like in the speech condition, the higher MMN mean amplitude in perceiving within-category deviants was evoked in the AM group than the NM group in the non-speech condition. This could be the result of increased demands for the acoustic analysis of pitch in the non-speech condition (Xu et al., 2006), and amateur musicians had more experience with fine-grained musical pitch by playing orchestral instruments (Sluming et al., 2002; Vuust et al., 2012; Patel, 2014). It is believed that in the speech condition, lexical tones distinguish the semantic meanings of words at a higher lexical level, and hence, phonological representations are exploited to a larger extent than acoustic processing by native Mandarin listeners; however, in the non-speech condition, the internal acoustic analysis for tone analogs tends to be implemented (Xu et al., 2006; Peng et al., 2010; Xi et al., 2010). As proposed by Wang (1973), lexical tones and segments, including nuclear vowels and optional consonants, are compulsory elements of Mandarin syllables. The lack of segments led pure tones to be non-speech signals, even though pure tones were interpolated with congruent cues of pitch, intensity, and duration to lexical tones. From this perspective, the perception of pure tones was largely contingent on the processing of pitch information, thereby in the non-speech condition, amateur musicians also showed improved performance in the processing of within-category deviants than non-musicians.

As regards categoricality of lexical tone perception, only non-musicians were observed to provoke the significantly larger MMN mean amplitude in the processing of across-category deviants than within-category deviants, suggesting that amateur musicians were less categorical than non-musicians in the current study. This was in partial agreement with a recent study which showed that Mandarin-speaking musicians do not consistently perceive native lexical tones more categorically than non-musicians (Chen et al., 2020). Based on a latest study by Maggu et al. (2021) that focused on absolute pitch (AP, the ability to name or produce a pitch without a reference) and found that listeners with AP are more sensitive to both across-category and within-category distinctions of lexical tones compared to their non-AP counterparts, our AM members might be non-AP listeners whereby they did not outperform NM in the processing of across-category deviants. This also complies with the study by Levitin and Menon (2003) demonstrating that it is very rare for individuals to have AP when they started musical experience after age 6. Crucially, the insignificant group difference of amplitudes in perceiving across-category deviants uncovered the dominant higher-level influences of linguistic categories relating to lexical tones (Zhang et al., 2011; Zhao and Kuhl, 2015b; Si et al., 2017). Zhao and Kuhl (2015b) found that Mandarin musicians’ overall sensitivity to lexical tones links with musical pitch scores, suggesting lower-level contributions; however, Mandarin musicians’ sensitivity to lexical tones along a continuum remains analogous to non-musicians. In the study by Wu et al. (2015), no group difference was observed in terms of across-category discrimination accuracy and peakedness in the discrimination function between Mandarin musicians and non-musicians when processing lexical tones. Similar to the studies mentioned above, both groups in the present study were already tonal-language experts and the phonetic inventories for the native language had been acquired and refined early in their lives (Zhang et al., 2005). In other words, as revealed by prior studies (Kuhl, 2004; Best et al., 2016; Chen F. et al., 2017), our listeners had developed robust linguistic representations before their inception of musicianship, which was consequently resistant to plastic changes driven by music (Besson et al., 2011a,b; Tang et al., 2016). Therefore, we had not tracked any clues to mirror that amateur musicians were augmented in tonal representations. This also echoes a study researching segmental vowel perception, which showed that musicians were not advantageous in identifying native vowels and thus they had no strengthened internal representations of native phonological categories in comparison to the non-musician counterparts (Sadakata and Sekiyama, 2011).

In the current study, the AM group outperformed the NM group in the perception of within-category deviants regardless of being speech or non-speech, indicating their superior abilities in pitch processing across domains, in line with Chandrasekaran et al. (2009). This speculation provides novel but persuasive support for OPERA-e (Patel, 2014) in that musical-pitch extends to lexical-pitch detection, even when derived from amateur musical experience. The facilitative pitch processing could be explained by taking into account the differences in pitch precision between music and lexical tones. According to OPERA-e, pitch variations in music can be smaller in frequency size than that in language, thereby pitch precision from music plays a profitable role in perceiving within-category lexical tone stimuli (Patel, 2014). Previous studies identified that a pitch interval as small as one semitone remains perceptually salient in music. For example, a C versus a C# in the key of C can be explicitly discerned (Patel, 2014; Tang et al., 2016). Nonetheless, for categorical perception of lexical tones, the smallest frequency range for discrimination is about 4–8 Hz for normal Chinese speakers in light of just-noticeable differences (JNDs, Liu, 2013). Pitch threshold is important in lexical tone perception. Amusia is a musical-pitch disorder influencing both music and speech processing (Peretz et al., 2002, 2008; Tillmann et al., 2011; Vuvan et al., 2015). Tone agnosics, a subgroup of individuals with amusia (Nan et al., 2010), struggle to perceive fine-grained lexical tones because the elevated pitch threshold ranges from 20 to 30 Hz (Huang et al., 2015a,b), which results in their impoverished performance as compared to typical listeners in categorical perception of lexical tones (Zhang et al., 2017). In the current study, as measured via Praat, the tonal stimuli were about 9 Hz distant for every step along the continuum of Mandarin T2–T4, and both deviants (S3 and S11) were four steps apart from the standard (S7). The frequency size of stimuli in the present study was far larger than that in music and JNDs. Thus, within-category pitch differences were detected more easily by amateur musicians than non-musicians.

However, other possibilities for enhanced within-category pitch perception should be acknowledged. Although the present sample demographic characteristics were well-controlled (Ayotte et al., 2002; Patel, 2014; Tang et al., 2016), the capacities of lexical tone perception in the AM participants before they started their musical experience were unknown. In other words, some participants might be sensitive to pitch information prior to their musical experience. Longitudinal studies with pre- and post-tests are thus highly recommended to further estimate the effects of amateur musical experience on speech processing. Some heritable differences in auditory functions should also be cautiously controlled (Drayna et al., 2001), since there exist naturally occurring variations in pitch perception capacities (Moreno et al., 2008; Qin et al., 2021). Meanwhile, although the overall gender ratio was nearly equal, different males and females were found between AM and NM groups. Previous studies claim that there are no gender effects in amplitude and latency of MMN among male and female participants (Kasai et al., 2002; Ikezawa et al., 2008; Tsolaki et al., 2015; Yang et al., 2016), while others hold an opposite position (Aaltonen et al., 1994; Barrett and Fulfs, 1998). Future studies should try to exclude the inconclusive effects by gender.

### Earlier Processing of Phonological Information Than Acoustic Information of Lexical Tones by Mandarin Listeners

The results showed that in both groups, across-category deviants elicited the significantly shorter MMN peak latency than within-category deviants, suggesting that phonological processing precedes acoustic processing for lexical tones. Note that unlike MMN mean amplitude, the two groups showed comparable MMN peak latency as revealed by the significant main effect of Deviant type. No group difference in terms of MMN peak latency might be ascribed to musical experience; in other words, the AM participants were not expert musicians, which mediated their abilities of pitch processing. For this reason, together with MMN mean amplitude and peak latency, the findings possibly suggest that the effects of amateur musical experience on lexical tone perception are somehow constrained. Therefore, only if musical expertise reaches the professional level, then a significant difference between the two groups in terms of latency can be anticipated. This tentative speculation needs to be further elaborated in future studies.

The current results relating to MMN peak latency provide counter-evidence to the findings by Luo et al. (2006) concerning the two-stage model. According to Luo et al. (2006), acoustic and phonological information about lexical tones are processed at pre-attentive and attentive stages, respectively. Nevertheless, many recent studies have shown that both acoustic and phonological information might be processed at pre-attentive and attentive stages in parallel (Xi et al., 2010; Yu et al., 2014, 2017). In accordance with these studies, the present results revealed that across-category stimuli were processed earlier than within-category stimuli, indicating that phonological information was processed ahead of acoustic information at pre-attentive stage, which differed from that proposed in the serial model. It is worth noting that the mentioned studies (Luo et al., 2006; Xi et al., 2010; Yu et al., 2014) recruited non-musician participants with similar neurocognitive mechanisms for processing lexical tones. Although the findings from Yu et al. (2014, 2017) comply with the notion that phonological information can be processed earlier than acoustic information, the current study further shows that this pattern of temporal processing occurs regardless of listeners’ musical background. In contrast to the studies using only non-musicians as participants, amateur musicians in the current study performed similarly to professional musicians showing advantages in processing within-category deviants, suggesting their enhanced processing of acoustic information (Wu et al., 2015; Chen et al., 2020). However, analogous to non-musicians, these amateur musicians still elicited the significantly longer MMN peak latency for within-category deviants than across-category deviants. This indicated that irrespective of the strengthened processing of acoustic information, phonological information was processed earlier than acoustic information at the pre-attentive cortical stage, as opposed to the two-stage model (Luo et al., 2006); besides, it confirmed the dominant role of higher-level linguistic categories relating to lexical tones from a neural perspective (Zhao and Kuhl, 2015b; Si et al., 2017). This provides the meaningful insight to the neural mechanisms which underlie the perceptual processing of lexical tones.

Presumably, some factors contributing to the current results of latency are worthy of consideration. First, the earlier processing of across-category than within-category deviants might be attributable to the properties of MMN. Näätänen (2001) illustrated that in auditory presentation, the differences between several infrequent deviant stimuli embedded in a flow of frequent and repeated standard stimuli can be automatically detected as signaled by MMN, with stronger incongruity leading to shorter latency onset. In the current study, across-category stimuli (S3) diverged from the standard (S7) in both phonological information of categories and acoustic information of pitch, while within-category stimuli (S11) only differed in acoustic information. Therefore, a shorter MMN peak latency was evoked by S3 than S11. Second, it is argued that tonal representations influence lexical tone processing (Xu et al., 2006; Chandrasekaran et al., 2007a; Chen S. et al., 2017). Although pure tones were non-speech sounds, the perception of them might be facilitated as a function of long-term phonological memory traces for lexical tones (Chandrasekaran et al., 2007a; Kraus and White-Schwoch, 2017). As explicated in Yu et al. (2014), the activation of long-term memory traces means that phonological information relating to lexical categories has some effects on the pitch detection of non-speech analogs, which copy identical acoustic cues from lexical tones. In the current study, the perception of pure tones as non-speech analogs to lexical tones might be impacted by memory traces for lexical tones, through which across-category deviants also elicited shorter latencies than within-category deviants in the non-speech condition regardless of group.

Some researchers have addressed that pitch type influences the temporal processing of lexical tones. As demonstrated in previous studies, T2 and T3 are acoustically similar such that their perception even burdens native speakers (Shen and Lin, 1991; Hao, 2012). Accordingly, Chandrasekaran et al. (2007b) found that MMN peak latency of T1–T3 is shorter than that of T2–T3 in Mandarin. Chandrasekaran et al. (2007b) concluded that MMN peak latency can be impacted by pitch type and likewise, the study by Yu et al. (2017) systematically investigated pitch type and latency, showing that pitch height is always processed ahead of pitch contour. The current study revealed that across-category deviants (S3) were processed earlier than within-category deviants (S11) with S7 being the standard. As shown in Figure 1, the contours of S3 versus S7 were more different than S11 versus S7 in slope, with a larger interval at onset point in terms of pitch height. In this regard, the differences in across-category deviants could be detected earlier, thus eliciting a shorter MMN peak latency in contrast to within-category deviants (Yu et al., 2017). This finding adds a further line of evidence supporting that pitch type is associated with MMN peak latency (Chandrasekaran et al., 2007b; Yu et al., 2017).

### Re-categorization of Participants’ Musicianship in Tests of Pitch Processing

From the methodological perspective, the results mentioned above require us to re-consider the categorization of participants’ musical experience. First, the current study emphasizes the importance of characterizing participants in light of their musical experience, because individuals with musical experience tend to outperform their non-musician counterparts in this field of research. Second, there have been various choices to select non-musicians without a conventional standard. For instance, non-musicians’ musical practice ranges differently from 0 to 3 years in previous studies (e.g., Alexander et al., 2005; Wong et al., 2007; Maggu et al., 2018). Besides, individuals with non-professional musical training may be unsuitably regarded as non-musicians (Shen and Froud, 2016, 2019), and even reporting musical background has been occasionally neglected in some studies of lexical tone processing (Hao, 2012; Morett, 2019; Qin et al., 2019). The neural evidence provided by the current study showed that similar to professional musicians, amateur musicians as non-music majors with around 4-year musicianship are strengthened in acoustic processing of lexical tones. Those listeners with a limited duration of musical experience (e.g., 3 years) might have already been affected regarding their pitch-detection abilities; hence, the criteria for screening non-musicians in the future should be stricter than in the past. Moreover, although we used the comparative approach to analyze lexical tone processing by amateurs and experts from previous literature, it is recommended to directly recruit one more group of professional musicians so as to systematically research the effects of magnitudes of musical expertise on speech processing.

The present results verified that amateur musical experience modulates categorical perception of lexical tones for native adults (i.e., enhanced within-category but comparable across-category lexical tone processing) though they have preexisting long-term tone language experience. In accordance with previous studies (Gordon et al., 2015; Zhao and Kuhl, 2015a,b), the current study supports the conceptual framework of OPERA-e by highlighting that the perceptual demands required for musical practice benefit the neural systems that are crucial for speech perception (Patel, 2014). Findings also echo those of previous studies indicating that music can be applied to prompt language skills in both normal and clinical populations due to the facilitative effects from music impacting on language (Won et al., 2010; Herholz and Zatorre, 2012; Petersen et al., 2015; Gfeller, 2016).

Since Duncan et al. (2009) identified that the mean amplitudes of the ERP components link with the volume of neural resources engaging in brain activities, future studies are advised to continue researching hemispheric processing of lexical tones by amateur musicians^2^. Note that for the analysis of lateralization among future studies, variance of neural data should be reduced by handedness, given that there is a strong bias of handedness on cerebral lateralization and left-handers may show anomalous dominance patterns (Cai and Van der Haegen, 2015; Plante et al., 2015). Moreover, conducive to generating some ecological impacts, these findings might also encourage individuals to engage in music in either formal or informal ways, thus aiding their aesthetic development as well as helping protect against cognitive decline (Román-Caballero et al., 2018). In addition, as validated by Näätänen et al. (2004) and Pakarinen et al. (2007), the MMNs obtained via the multifeature passive oddball paradigm were equal in amplitude to those via the traditional MMN paradigm. However, it should be treated with caution when calculating MMNs, because different endogenous ERPs would be generated by this multifeature passive oddball paradigm. Due to the potential for physical confounds existing among auditory stimuli, MMNs can be obtained by subtraction from a given sound when it is a standard to the exact same sound when it is a deviant (Schröger and Wolff, 1996). Future studies are encouraged to tap this paradigm in experiments. Lastly, as an important auditory ERP component, MMN can provide an objective marker to measure the abilities of amateur musicians to discriminate lexical tones.

## Conclusion

In summary, the current study explored cortical plasticity among adult amateur musicians, taking advantage of neurophysiological MMN indices. Although participants were native speakers of Mandarin, the results of the MMN mean amplitude indicated that the abilities to process acoustic information by amateur musicians were enhanced in terms of categorical perception of Mandarin lexical tones. Higher sensitivity for pitch shifts across domains confirmed that speech perception can be modulated by amateur musical experience in adulthood, and music associates with language even only an amateur level of musical expertise is reached by listeners. This indicated that the advantageous effects of music on speech processing are not restricted to a small group of professional musicians but extend to a large population of amateur musicians. In addition, a shorter latency was evoked by across-category deviants than that by within-category deviants, suggesting that these two types of information can be processed concurrently at the pre-attentive cortical stage; more precisely, the processing of phonological information is earlier than that of acoustic information, even for amateur musicians whose acoustic processing was strengthened for lexical tones.

## Data Availability Statement

The original contributions generated for this study are included in the article/supplementary material, further inquiries can be directed to the corresponding author.

## Ethics Statement

The studies involving human participants were reviewed and approved by the Ethics Review Board at the School of Foreign Languages of Hunan University. The patients/participants provided their written informed consent to participate in this study.

## Author Contributions

JZ and XC contributed to the conception of the study. JZ conducted the experiments and drafted the manuscript. XC and YY contributed to the revision of the manuscript. All authors have approved the final version of the manuscript.

## Conflict of Interest

The authors declare that the research was conducted in the absence of any commercial or financial relationships that could be construed as a potential conflict of interest.

## Footnotes

1 The way of Collapsed Localizers has been introduced in Luck and Gaspelin (2017), depicting that the researchers average the waveforms across conditions and then use the timing and scalp distribution from the collapsed waveforms to define the analysis parameters for the non-collapsed data.

2 Following Xi et al. (2010), we compared amplitudes of F3 and F4 and added this as an extra within-subject factor Electrode to probe into the hemispheric pattern, which indicated that the main effect or interactions of Electrode with other factors were not significant (*p*s > 0.1). Despite the low spatial resolution of ERP, the result as a preliminary sign manifested that the perception of Mandarin lexical tones is supported by neither one specific area nor a single hemisphere (Gandour et al., 2004; Witteman et al., 2011; Price, 2012; Si et al., 2017; Liang and Du, 2018; Shao and Zhang, 2020).
